# Supplementary material for: Phytohormone Production by the Endophyte Bacillus safensis TS3 Increases Plant Yield and Alleviates Salt Stress
Source: Plants (Basel). 2023 Dec 26;13(1):75. doi: 10.3390/plants13010075 (PMC10780329; doi:10.3390/plants13010075)
Supplement: Supplementary file 1 [file plants-13-00075-s001.zip › plants-2732819-supplementary.pdf]

Supplementary Materials

Table S1. Agrochemical characteristics of the soil of the potato experimental site

pH_{KCl}	Sum $N\text{-}NO_3$ $N\text{-}NH_4$	P_2O_5	K_2O	Humus
	mg/kg of soil	mg/kg of soil		%
4.95	24.5	342	64	1.7

Table S2. Agrochemical characteristics of the soil of the white cabbages experimental site

pH_{KCl}	Sum $N\text{-}NO_3$ $N\text{-}NH_4$	P_2O_5	K_2O	Humus
	mg/kg of soil	mg/kg of soil		%
6.1	9.0	472	167	1.62
